# Genome-Wide Identification, Characterization and Function Analysis of Lineage-Specific Genes in the Tea Plant *Camellia sinensis*

**DOI:** 10.3389/fgene.2021.770570

**Published:** 2021-11-10

**Authors:** Zhizhu Zhao, Dongna Ma

**Affiliations:** Key Laboratory of the Ministry of Education for Coastal and Wetland Ecosystems, College of the Environment and Ecology, Xiamen University, Xiamen, China

**Keywords:** tea plant, lineage-specific genes, gene duplication, transcriptome, *Camellia*

## Abstract

Genes that have no homologous sequences with other species are called lineage-specific genes (LSGs), are common in living organisms, and have an important role in the generation of new functions, adaptive evolution and phenotypic alteration of species. *Camellia sinensis* var. *sinensis* (CSS) is one of the most widely distributed cultivars for quality green tea production. The rich catechins in tea have antioxidant, free radical elimination, fat loss and cancer prevention potential. To further understand the evolution and utilize the function of LSGs in tea, we performed a comparative genomics approach to identify *Camellia*-specific genes (CSGs). Our result reveals that 1701 CSGs were identified specific to CSS, accounting for 3.37% of all protein-coding genes. The majority of CSGs (57.08%) were generated by gene duplication, and the time of duplication occurrence coincide with the time of two genome-wide replication (WGD) events that happened in CSS genome. Gene structure analysis revealed that CSGs have shorter gene lengths, fewer exons, higher GC content and higher isoelectric point. Gene expression analysis showed that CSG had more tissue-specific expression compared to evolutionary conserved genes (ECs). Weighted gene co-expression network analysis (WGCNA) showed that 18 CSGs are mainly associated with catechin synthesis-related pathways, including phenylalanine biosynthesis, biosynthesis of amino acids, pentose phosphate pathway, photosynthesis and carbon metabolism. Besides, we found that the expression of three CSGs (*CSS0030246*, *CSS0002298,* and *CSS0030939*) was significantly down-regulated in response to both types of stresses (salt and drought). Our study first systematically identified LSGs in CSS, and comprehensively analyzed the features and potential functions of CSGs. We also identified key candidate genes, which will provide valuable assistance for further studies on catechin synthesis and provide a molecular basis for the excavation of excellent germplasm resources.

## Introduction

Genes that have no homologous sequences with other species are called lineage-specific genes (LSGs), sometimes are also called orphan genes (Fischer and Eisenberg, 1999; Tautz and Domazet-Loso, 2011). LSGs were first found in *Saccharomyces cerevisiae* in 1996, that is, a large number of genes in the genome showed no similarity to the database sequence, accounting for about 26% of the genome (Dujon, 1996). As more and more complete genomes and transcriptomes from different species have been sequenced, LSGs have also been more and more widely studied, from microorganisms to plants, such as legumes (Graham et al., 2004), *Triticeae* (Ma et al., 2020), *Oryza sativa* (Yang et al., 2009), *Arabidopsis* (Lin et al., 2010), Poaceae (Campbell et al., 2007), *Populus* (Yang et al., 2009) and sweet orange(Xu et al., 2015). The proportion of LSGs in different genomes is also different, and it has been found that the average proportion of LSGs in plants is higher than the average proportion in animals (Yang et al., 2013). The significance of the presence of most LSGs remains unknown, but is often associated with the unique features the species have and stress tolerance, which is of important implications for elucidating the evolutionary of species (Khalturin et al., 2009).

Although we cannot analyze the biological functions of LSGs using homology-based functional classification, the structural traits of their sequences may provide some initial clues for LSGs exploration. Compared with evolutionary conserved genes (ECs), LSGs have some differences in gene length, number of introns and exons, GC content and chromosome distribution preference, owing to the shorter generation time. LSGs are normally characterized by shorter gene length and fewer exons in eukaryotes (Domazet-Loso, 2003; Campbell et al., 2007; Toll-Riera et al., 2009; Yan et al., 2014). GC content of LSGs to most species is lower than that of ECs, a characteristic that is not universal. For example, the GC content of LSGs in zebrafish is higher than conserved genes, this characteristic is similar to the LSGs in rice (Yang et al., 2013). The distribution characteristics of LSGs on chromosomes are also different, like zebrafish have uneven distribution of LSGs on chromosomes, with some chromosomes having a high proportion of gene and others having no LSGs, which may be related to the length of chromosome (Yang et al., 2013). Nevertheless, the distribution of LSGs in *Arabidopsis* and ant has no chromosomal preference and LSGs are evenly distributed among non-LSGs throughout the genome (Donoghue et al., 2011; Wissler et al., 2013). In addition to sequence traits, some LSGs show a high degree of tissue-specific expression (Lemos et al., 2005). LSGs were more expressed in callus in sweet orange, a stem-cell like tissue (Xu et al., 2015) and most LSGs in wheat were expressed in sexual tissues (Ma et al., 2020).

Studies found that the expression of some LSGs responded to a wide range of stress conditions, suggesting that these LSGs may enable the species to better adapt to the environment, thus LSGs become important genes during evolution (Khalturin et al., 2009; Donoghue et al., 2011). LSGs in mangrove *Aegiceras corniculatum* are involved in pathways like flavonoid biosynthesis, which play a role in oxidative toxicity mitigating in mangrove plants under high saline environments (Ma et al., 2021). The LSG in *Arabidopsis thaliana QQS* was involved in regulating the partitioning of carbon and nitrogen among proteins and carbohydrates in leaves (Li et al., 2015). The rice orphan gene *OsDR10* was reported to enhance disease resistance by increasing endogenous salicylic acid (SA) levels and suppressing the production of endogenous jasmonic acid (JA) (Xiao et al., 2009). The overexpression of new gene *GS9* in rice results in round grains, which can be used to breed rice varieties with optimized grain shape (Zhao et al., 2018). A study conducted in six orphan genes in *Drosophila* showed that arbitrary suppression of four of these six orphan genes via RNAi caused lethality (Reinhardt et al., 2013). Obviously, LSGs are involved in different metabolic pathways and have diverse functions that affect various aspects of organisms, and the importance of LSGs is just showing up.

Tea is one of the most well known and most consumed beverages in the world, which provides both health benefits and economic value (Song et al., 2012; Lee et al., 2014; Zhu et al., 2020). Due to the benefits tea brings to health, the exploration of tea has increased at molecular level. Tea belongs to the Theaceae family, and is a quite important economically crop worldwide whose leaves can be used to produce various tea. Because of the variation of gene, the difference in growing conditions and the difference in processing modes, tea always has diverse palatability, like bitter, astringent, and sweet flavors (Song et al., 2012). *Camellia sinensis* var. *sinensis* (CSS) is one of the most widely distributed cultivars for quality green tea production (Song et al., 2012). Currently, 67% elite tea plant cultivars belong to CSS. During rapid evolution, LSGs are endowed with new biological functions when subjected to external environmental pressures, which allow species to better adapt to the external environment (Long et al., 2003; Kaessmann, 2010). In addition, some LSGs participate and play important roles in metabolic networks and pathways affecting various aspects of the organism soon after their origin (Chen et al., 2012), and thus making LSGs important genes during evolution. To further understand the evolution and utilize the function of LSGs in tea species CSS, we used a comparative genomics approach to identify *Camellia*-specific genes (CSGs) in the CSS genome for analyzing the origin models, structural properties and subcellular localization of the CSGs. Furthermore, we constructed weighted gene co-expression network analysis (WGCNA) to predict the function of LSGs in CSS. Collectively, these results will provide important information for understanding the role played by CSGs in the evolution of lineage specific phenotypes and adaptive innovation in CSS.

## Materials and Methods

### Data Collection

The predicted CSS high-quality genome annotation and the expression level genes from eight different tissues (apical bud, young leaf, mature leaf, old leaf, stem, flower, fruit and stem) were downloaded from http://tpdb.shengxin.ren/. We collected *A. chinensis* genomes from public databases to identify LSGs in CSS (ftp://bioinfo.bti.cornell.edu/pub/kiwifruit/)*.* Other 126 plant genome sequences were downloaded from Phytozome V13.1 (http://phytozome.jgi.doe.gov/pz/ portal.html) (Supplementary Table S1),the assembled unique transcripts (PUT) sequences of the plants were downloaded from PlantGDB (http://www.plantgdb.org/prj/ESTCluster/progress.php) (Supplementary Table S2), Uniprot-KB were downloaded from Uniprot (ftp://ftp.ebi.ac.uk/pub/ databases/uniprot/knowledgebase/) and NR databases were acquired from NCBI, respectively.

### Identification of CSGs

**FIGURE 1:**
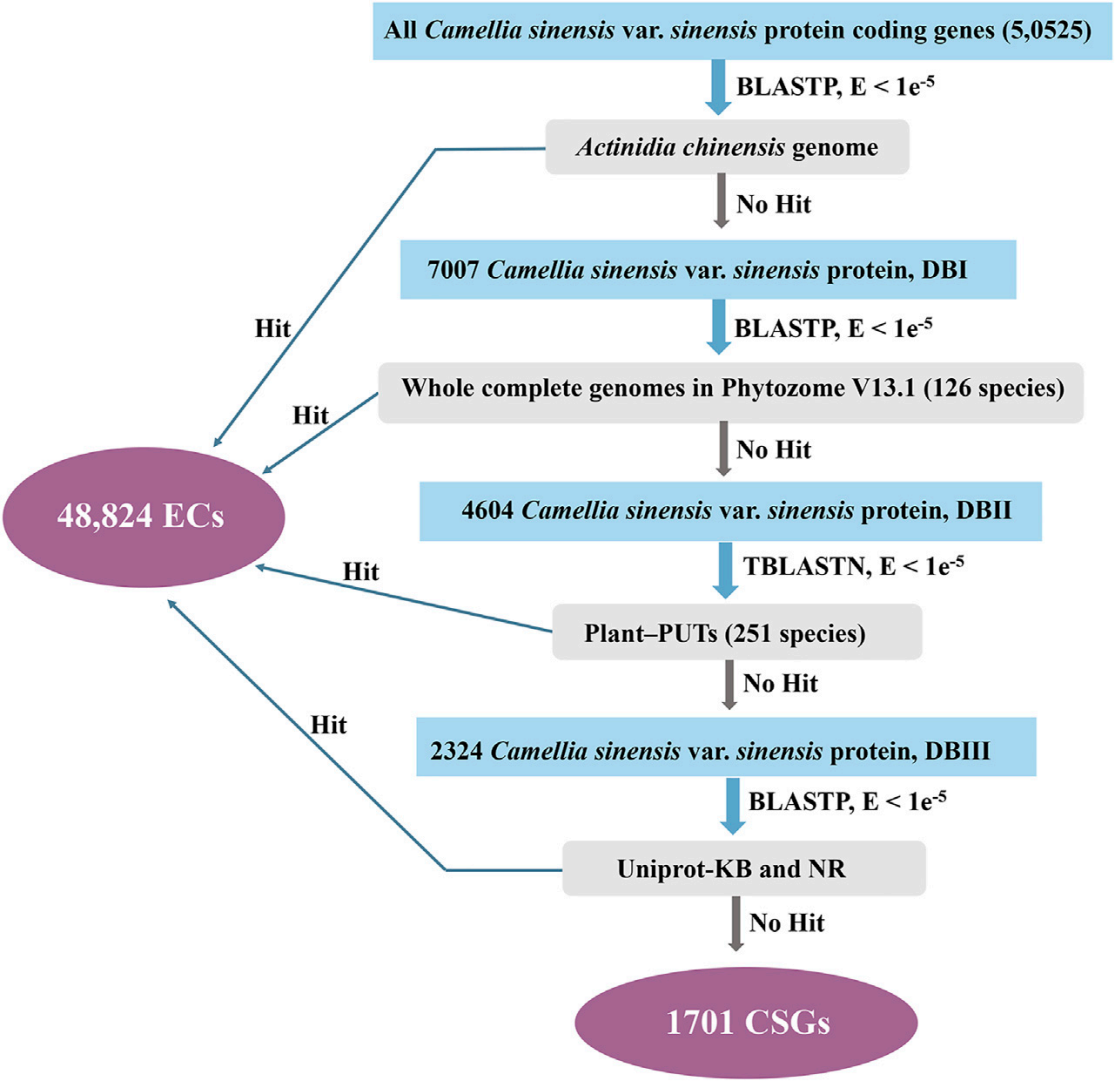
Procedure for identifying lineage-specific genes in *Camellia sinensis* var. *sinensis*.

The study on the origin and evolution of CSGs has been improved due to the development of comparative genomics. Based on a homolog search, CSGs within CSS were identified in a pipeline (Figure 1). Firstly, CSS protein sequences were searched against *A. chinensis* proteome data using BLASTP. The CSS protein sequence was discarded once it has BLASTP hit with an E-value cutoff of 1e-5. We then performed homology searches with genomes of other plants, Plant-PUTs database, Uniprot-KB database and NR database in turn with an E-value cutoff of 1e-5. Finally, the genes having no homolog to any databases are the CSGs (Zhang et al., 2007; Lin et al., 2010), while the others which are homologous are evolutionarily conserved genes (ECs).

### Genic Features

We used the whole genome information of CSS to observe the structural characteristics of the CSGs. The isoelectric point of CSGs and ECs was measured using DAMBE7 software (Xuhua, 2018). Differences between CSGs and ECs, including gene size, length of protein, size of the exons and introns, number of the exons and content of GC were calculated using the in-house python scripts. The significant difference between different groups of CSGs and ECs was then determined with Mann-Whitney U test. We extracted the information of chromosome localization from chromosome sequences and mapped it with MapGene2Chrom (http://mg2c.iask.in/mg2c_v2.0/), and finally predicted CSGs subcellular localizations using BUSCA (Bologna Unified Subcellular Component Annotator) (Savojardo et al., 2018)**.**

### Gene Duplication Analysis

There are multiple models explaining for the origin of LSGs according to previous studies (Wu et al., 2011; Wissler et al., 2013), among which gene duplication has long been thought as the primary mechanism of the emergence of LSGs (Zhang, 2003). We first searched for homologous genes with an E-value cutoff of 1e-8 using BLASTP, and then used DupGen_finder.pl to determine the different types of gene duplication, which is able to identify WGD, tandem duplication, proximal duplication, transposon duplication and dispersed duplication (Qiao et al., 2019). The *Ks* of the duplication paralogous gene pairs were computed with the python script synonymous_calc.py (https://github.com/tanghaibao) with the method of Nei-Gojobori. We finally estimated the time of gene duplication of CSGs with the universal mutation rate of 6.5 × 10^–9^ (Gaut et al., 1996).

### Gene Expression Analysis

To analyze the environmental adaption of CSGs, we downloaded the transcriptome data of CCS from the European Nucleotide Archive database (ENA; http://www.ebi.ac.uk/ena) under project number accession PRJEB11522. Plants in this experiment were divided into three groups, the treatment of the first group was 25% percent polyethylene glycol to simulate drought stress conditions, the second group was 200 mM NaCl, and the last group was a blank control with sampling times at 0, 24, 48, and 72 h (Zhang et al., 2017). We then used Trimmomatic program to filter the raw RNA-seq data (Bolger et al., 2014). In order to identify differentially expressed genes (DEGs) among different treatments, the abundance_estimates_to_matrix.pl, run_DE_analysis.pl (edgeR) and analyze_diff_expr.pl modules of the Trinity package with default settings were used. The |log2FC| ≥ 1 and a false discovery rate (FDR) < 0.05 as the threshold were implied to determine the significant differences in gene expression, and RSEM implemented in Trinity package was applied to compute FPKM (fragments per kilobase of exon per million fragments mapped) (Grabherr et al., 2011). Based on RNA-seq data, cluster analysis was performed with R software, and the specific expression of the genes were selected for follow-up functional validation. Genes with FPKM value >0.02 were assumed to have been expressed (Ma et al., 2020). In addition, the genes specifically expressed in certain tissue were identified using PaGeFinder software with specificity measure (SPM) (Pan et al., 2012), and it was identified as a specific gene in this tissue once the SPM value was ≥0.9.

### Weighted Gene Co-Expression Network Analysis and Function Annotation

After discarding the genes with FPKM <1, we then constructed WGCNA and divided these genes into modules with the help of WGCNA package in R software (Langfelder and Horvath, 2008). The network was built with default parameters using the automatic network builder function block wise Modules. We then calculated the eigengene value for each module in each tissue, and selected the module owing highest correlation coefficient while satisfying *p*-value < 0.05 as the tissue-specific module for further analysis. The most representative gene in each module was considered to be the module eigengene (ME). Module membership (MM) and gene significance (GS) of each ME were calculated in each tissue-specific module, and once MM > 0.95 and GS > 0.85 were satisfied, this gene was considered as a hub gene of this module. KEGG enrichment analysis was performed on an online platform, OmicShare (https://www.omicshare.com/).

## Results

### Identification of CSGs

Using the database resources released recently, CSGs in CSS were identified based on methods used in previous studies (Figure 1) (Toll-Riera et al., 2009; Yang et al., 2009; Lin et al., 2010; Tautz and Domazet-Loso, 2011). In this study, there were 50,525 annotated protein-coding genes within CSS genome in all, the they were used to perform BLASTP with Theaceae family genome (*Actinidia chinensis*) that had already been published. In this step, a total of 43,518 CSS genes had significant similarity (E-value < 1 e-5) and 7007 genes (DBⅠ) were retained for subsequent analysis. We removed the ECs showing homology and further searched the remaining genes against 126 plant genomes from Phytozome v13.1, resulting in 4604 genes retained for the next step of searches (DBⅡ). In the following comparison of these 4604 genes with 251 PlantGDB-assembled Unique Transcripts (PUTs) sequences, 2324 genes could not find any homologs (DBⅢ). Finally, to further eliminate the effect of false positives on the analysis, the remained genes were analyzed against the UniProt-KB and NR databases, a step that ultimately left 1701 genes. We termed these last remaining 1701 genes as CSGs in the CSS genome, making up 3.37% of the whole CSS genome (Supplementary Table S3), while these remaining 48,824 genes with similarities in the database were defined as ECs.

### High Proportion of CSGs Generated *via* Gene Duplications

**FIGURE 2:**
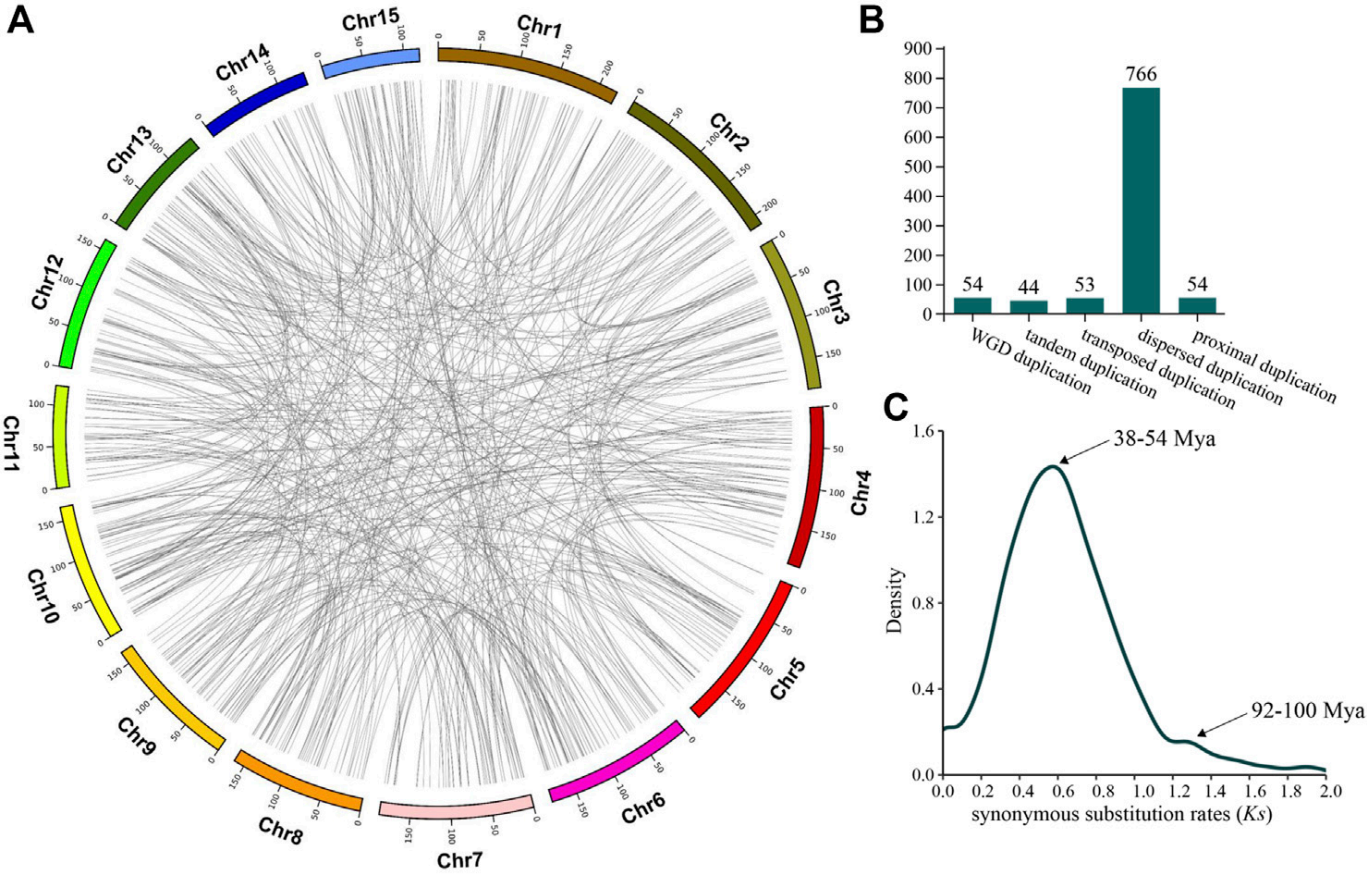
*Camellia*-specific genes (CSGs) originating from gene duplication. **(A)** Distribution of CSGs on chromosomes by five mechanisms of origin. **(B)** The CSGs number of different duplication types. **(C)** Density distribution of synonymous substitution rates (*Ks*) values between CSGs and paralogous genes.

There are several mechanisms regarding how LSGs were created, among which gene duplication has been long considered to be a major way for the origin of LSGs, and the creation of a new gene in the gene duplication model originates mainly through the differentiation after duplication. In this experiment, of the 1701 CSGs we identified from the genome of CSS, 971 CSGs originated from gene duplication, representing 57.08% of all CSGs (Supplementary Table S4) and evenly distributed on each chromosome (Figure 2A). Among the CSGs originating from gene duplication, a total of 54 CSGs were detected to create during WGD duplication. Besides, the number of CSGs generated by tandem duplication, transposed duplication, proximal duplication and dispersed duplication were 44, 53, 54, and 766 (Figure 2B), respectively. Obviously, CSGs were mainly produced by gene duplication. We used synonymous substitution rates (*Ks*) to assess the timeline for the gene duplication to occur in CSGs. As a result, there were two peaks, one with *Ks* = 0.5—0.7 and a second with *Ks* = 1.2—1.3 (Figure 2C), corresponding to the duplication time of 38–54 and 92–100 million years ago (MYA).

### Features of CSGs

**FIGURE 3:**
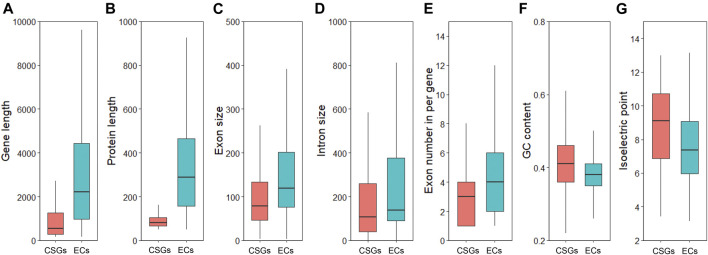
Analyze and compare the structural characteristics of *Camellia*-specific genes (CSGs) and evolutionarily conserved genes (ECs). **(A)** Box-plot comparisons of gene length. **(B)** protein length. **(C)** exon size. **(D)** intron size. **(E)** exon number in per gene. **(F)** GCs content. **(G)** isoelectric point.

To clarify whether there were significant differences between CSGs and ECs, we focused on analyzing and comparing the sequence structural features between the 1701 CSGs and 48,824 ECs identified in this study. As a result, both gene size (Figure 3A) and protein length (Figure 3B) of CSGs were significantly smaller compared to ECs (Table 1), with 1484.21 bp for CSGs gene size and 92.19 amino acids (aa) for CSGs protein length, 5367.51 bp for ECs gene size and 370.21 aa for ECs protein length (Table 1). The exon size (Figure 3C) and intron size (Figure 3D) of CSGs were both smaller than those of ECs, and the number of exons per gene of CSGs was also significantly less than ECs (Figure 3E). GC content in gene (Figure 3F), CDS and exon of CSGs were all significantly higher (Table 1). In addition, the isoelectric point was 8.65 for CSGs and 7.51 for ECs, which was obviously higher for CSGs. (Figure 3G; Table 1). Overall, the result indicated that there were obvious differences between CSGs and ECs in genetic features.

**TABLE 1:** Genic features of *camellia*-specific genes (CSGs) compared with evolutionarily conserved genes (ECs).

Items	CSGs		ECs		Mann-whitney U test Probability
	Mean (SE)	Median	Mean (SE)	Median	
Gene size (bp)	1484.21 (333.02)	573	5367.51 (7663.03)	2825	<0.0005
Protein length (aa)	92.19 (39.37)	80	370.21 (290.56)	301	<0.0005
Exons per gene	2.77 (1.68)	3	5.18 (4.52)	4	<0.0005
Exon size (bp)	106.77 (148.83)	79	247.87 (346.23)	134	<0.0005
Intron size (bp)	584.06 (1782.77)	140	942.51 (2198.02)	229	<0.0005
Gene GC content (%)	41.60 (0.67)	40.74	38.78 (5.17)	37.56	<0.0005
CDS GC content (%)	45.96 (0.55)	45.56	44.78 (4.30)	44.13	<0.0005
Exon GC content (%)	44.51 (0.88)	44.64	43.22 (0.6)	42.92	<0.0005
Isoelectric point	8.65 (2.44)	8.65	7.51(1.96)	7.36	<0.0005

**FIGURE 4:**
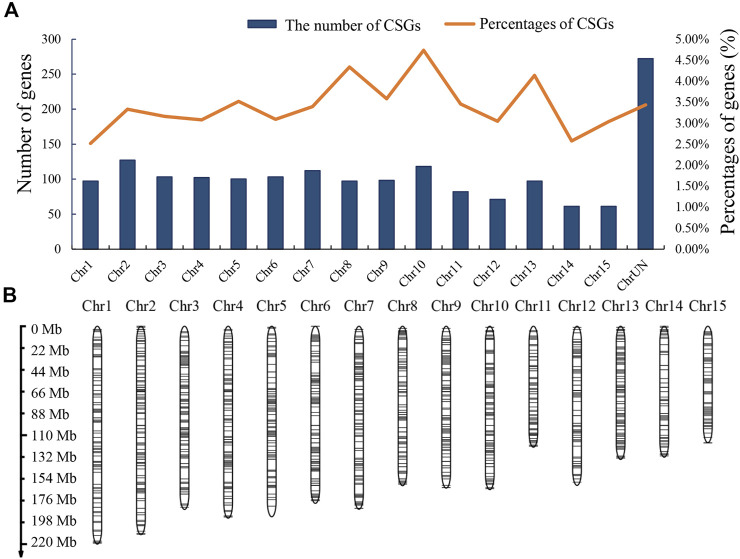
*Camellia*-specific genes (CSGs) distribution on chromosomes. **(A)** The numbers of CSGs on each chromosome of *Camellia sinensis* var. *sinensis*. Both numbers and percentages are shown. **(B)** Chromosomal distribution of the identified CSGs. Black horizontal lines represent CSGs.

To analyze the genomic distribution of CSGs, we mapped the CSGs on the chromosomes of CSS (Figure 4A) according to the information annotated in the genome (Supplementary Table S3). In total, there were 1429 CSGs distributed on 15 chromosomes. The highest number of CSGs on each chromosome was Chr2 (127), Chr7 (112) and Chr10 (118) in that order, while the highest percentage of CSGs on each chromosome was Chr10 (4.74%) and Chr8 (4.34%). It was clear that CSGs showed a preferential distribution on some chromosomes compared to ECs. In addition, the distribution of CSGs was more balanced on chromosomes except for the regions close to the telomeres of chromosome Chr5, Chr6, and Chr15 where the distribution of CSGs was sparse (Figure 4B). Overall, CSGs were relatively evenly distributed on these 15 chromosomes.

### Subcellular Localization

**FIGURE 5:**
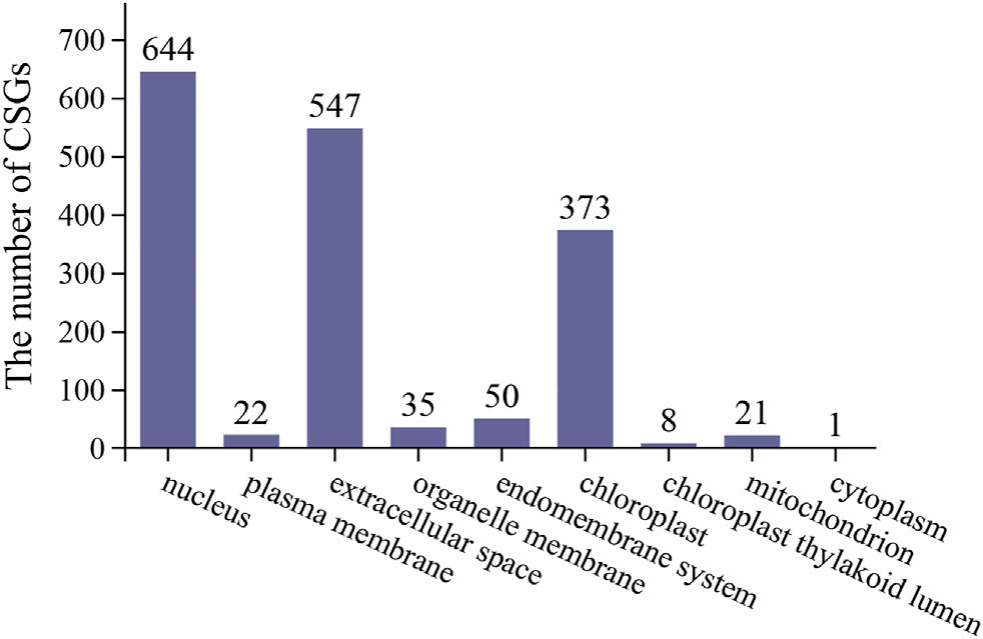
*Camellia*-specific genes assigned to different subcellular locations.

The function of proteins can usually be inferred to some extent based on their subcellular localization. Of the 1701 CSGs identified in this study, 644 were localized in the nucleus, 547 in extracellular space, 373 in chloroplast, 50 in endomembrane system, 35 on organelle membrane, 21 on mitochondria, 22 on the plasma membrane, eight on chloroplast thylakoid lumen, and only one on cytoplasm (Figure 5).

### Expression Profiles of CSGs

**FIGURE 6:**
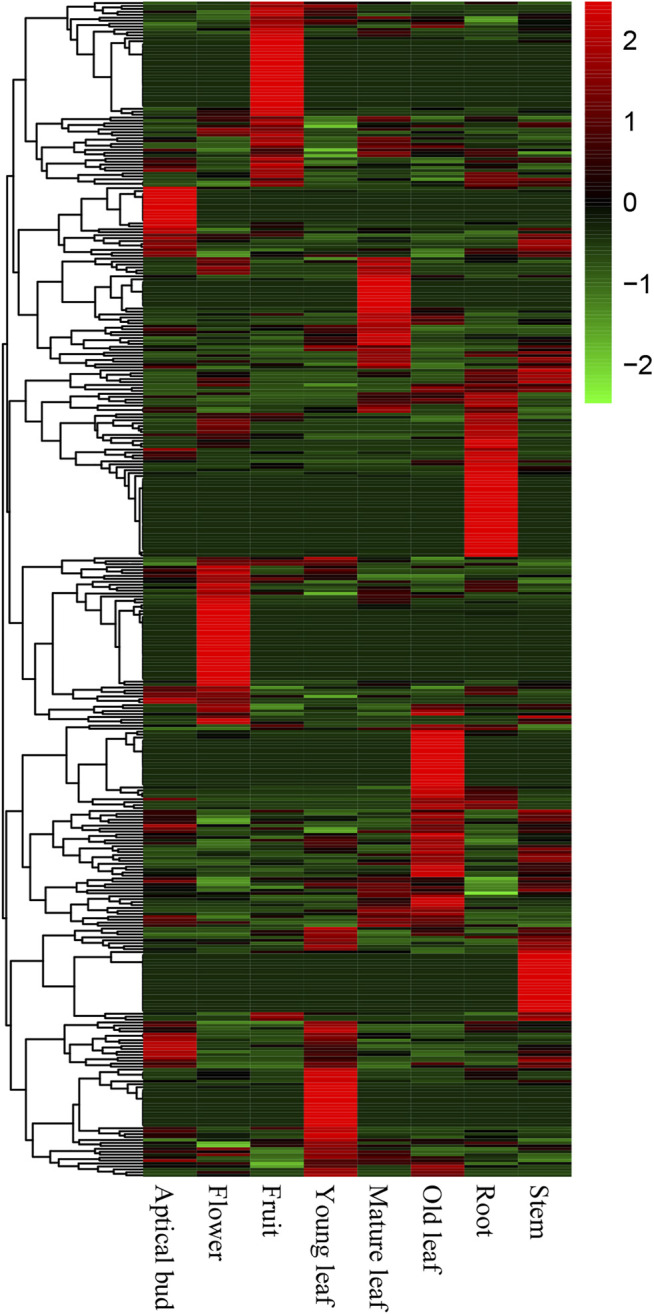
Expression pattern of *Camellia*-specific genes (CSGs) in different tissues includes apical bud, young leaf, mature leaf, old leaf, stem, flower, fruit, and root.

The expression pattern of a gene in different tissues is crucial to elucidate whether this CSG has a corresponding biological function. We downloaded RNA-seq data from eight tissues of CSS. The transcriptional data contained 400 (23.52%) CSGs and 40,897 (83.76%) ECs with FPKM >0.02. Among them, 194 CSGs were found to be expressed in all eight tissues (FPKM >2 in a minimum of one tissue), and 16 CSGs were shown to be highly expressed in all eight tissues (FPKM >2 in all of them) (Table 2). Based on the expression abundance in each tissue, it can be seen that most CSGs are expressed with tissue preference (Figure 6). Further studies found, 212 CSGs showed specific expression in eight tissues, of which 17 were specifically expressed in apical bud, 21 in young leaf, 18 in mature leaf, 28 in old leaf, 25 in stem, 32 in flower, 29 in fruit and 42 in root (Table 2; Supplementary Table S5), these genes might play unique roles in the corresponding tissues. Besides, a total of 6589 ECs was identified, of which 487, 416, 505, 33, 582, 1855, 525, and 2186 were specifically expressed in apical bud, young leaf, mature leaf, old leaf, stem, flower, fruit and root, respectively (Table 2). It was clear that CSGs (53%) were more likely to express in specific tissues than ECs (16.11%).

**TABLE 2:** Tissue expression pattern of *camellia*-specific genes (CSGs) and evolutionary conserved genes (ECs).

Items	Apical bud	Flower	Fruit	Young leaf	Mature leaf	Old leaf	Root	Stem	Total
With tissue-specific expression									
Number of ASGs (%)	17 (8.02)	32 (15.09)	29 (13.68)	21 (9.91)	18 (8.49)	28 (13.21)	42 (19.81)	25 (11.79)	212 (100)
Number of ECs (%)	487 (7.39)	1855 (28.15)	525 (7.97)	416 (6.31)	505 (7.66)	33 (0.5)	2186 (33.18)	582 (8.83)	6589 (100)
With high expression abundance (FPKM >2)									
Number of ASGs (%)	64(10.96)	78(13.36)	72(12.33)	69(11.82)	75(12.84)	65(11.13)	84(14.38)	77(13.18)	584(100)
Number of ECs (%)	24742(13.02)	22251(11.71)	23643(12.44)	24265(12.77)	23830(12.54)	21315(11.22)	24370(12.82)	25639(13.49)	190055(100)

To explore the potential relationship between CSG and environmental adaptation, we analyzed the expression of CSGs under salt and drought stress. A total of 12 CSGs were found to be stimulated by environmental stress compared to CK treatment under the criteria of ≥1.5-fold expression differential and the false discovery rate (FDR) < 0.01. There were 5 and 4 CSGs responded to salt and drought, respectively (Supplementary Table S6). Surprisingly, among these genes, *CSS0030246*, *CSS0002298,* and *CSS0030939* responded to both types of stresses (Supplementary Table S6), suggesting that these three genes probably function importantly roles in stress tolerance.

### CSGs Function Prediction

**FIGURE 7:**
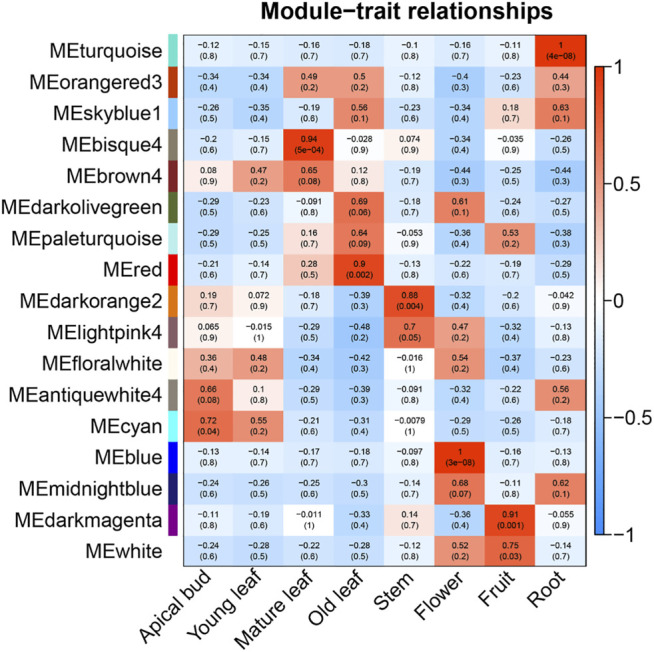
Heat map of module-tissue relationship.

**FIGURE 8:**
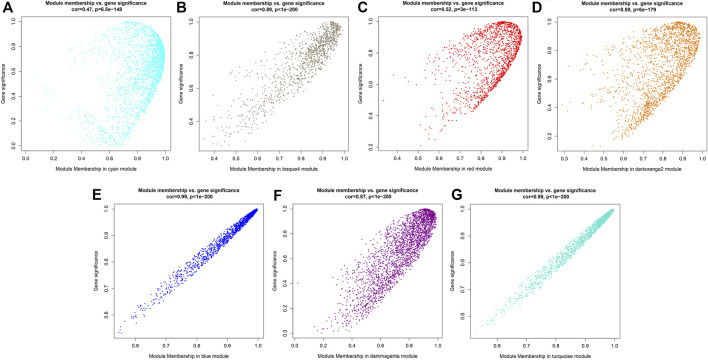
Gene significance map. **(A)** Gene significance map for members of module MEcyan. **(B)** Gene significance map for members of module MEbisque4. **(C)** Gene significance map for members of module MEred. **(D)** Gene significance map for members of module MEdarkorange2. **(E)** Gene significance map for members of module MEblue. **(F)** Gene significance map for members of module MEdarkmagenta. **(G)** Gene significance map for members of module MEturquoise.

**FIGURE 9:**
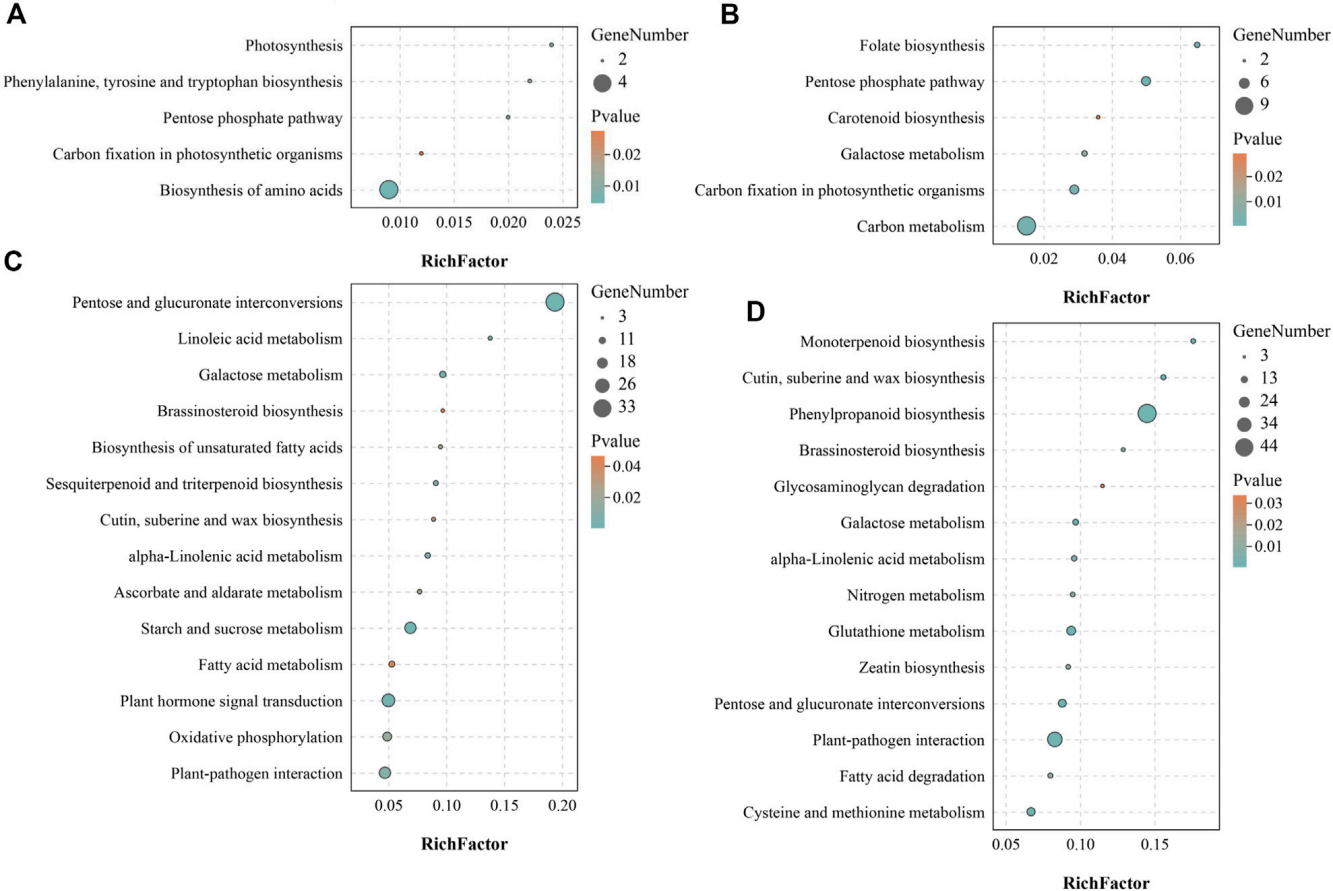
KEGG enrichment analysis of four tissue-specific modules. **(A)** KEGG enrichment analysis result of MEbisque4 module genes. **(B)** KEGG enrichment analysis result of MEred module genes. **(C)** KEGG enrichment analysis result of MEblue module genes. **(D)** KEGG enrichment analysis result of MEturquoise module genes.

Since it was impossible to infer the function of CSGs through homologous genes, but CSGs were specifically expressed in different tissues (Table 2). We used WGCNA, a method to identify synergistic gene modules, to further analyze the potential functions of CSGs in different tissues. We identified 17 modules. Treating different tissues as traits, we screened the most optimally correlated modules for the characteristic vector genes and phenotypes and plotted a heat map of module-trait relationships. We finally identified seven modules with extremely strong positive correlation with the trait, and the correlation coefficient (CC) between MEcyan module and apical bud reaches 0.72 (*p*-value = 0.04), MEbisque4 and mature leaf (CC = 0.94, *p*-value = 5 × 10^–4^), MEred and old leaf (CC = 0.9, *p*-value = 0.002), MEdarkorange2 and stem (CC = 0.88, *p*-value = 0.004), MEblue and flower (CC = 1, *p*-value = 3 × 10^–8^), MEdarkmagenta and fruit (CC = 0.91, *p*-value = 0.001) and MEturquoise and root (CC = 1, *p*-value = 4 × 10^–8^), respectively (Figure 7). The Pearson correlation coefficients (PCC) were calculated to derive seven tissue-specific modules (Figures 8A–G). We then identified 3187 hub genes in seven modules after screening (Supplementary Table S7), among them, including 18 CSGs. In MEbisque4 (mature leaf), there were 148 hub genes, including one CSGs. In MEred model (old leaf), there were 210 hub genes, including 2 CSGs. In MEblue model (flower), there were 1,108 hub genes, including 9 CSGs. In MEturquoise model (root), there were 140 hub genes, including 6 CSGs (Supplementary Table S8). These four modules were immediately subjected to KEGG enrichment analysis. In MEbisque4 (mature leaf), it is mainly enriched in biosynthesis of amino acids (ko01230), photosynthesis (ko00195), phenylalanine, tyrosine and tryptophan biosynthesis (ko00400), pentose phosphate pathway (ko00030) and carbon fixation in photosynthetic organisms (ko00710) (Figure 9A). In MEred model (old leaf), it is primarily affluent in pentose phosphate pathway (ko00030), carbon fixation in photosynthetic organisms (ko00710), folate biosynthesis (ko00790), carbon metabolism (ko01200), galactose metabolism (ko00052) and carotenoid biosynthesis (ko00906) (Figure 9B). In MEblue model (flower), pentose and glucuronate interconversions(ko00040), starch and sucrose metabolism (ko00500), galactose metabolism (ko00052), plant hormone signal transduction (ko04075) and alpha-Linolenic acid metabolism (ko00592) were enriched (Figure 9C). In MEturquoise model (root), it is mainly enriched phenylpropanoid biosynthesis (ko00940), plant-pathogen interaction (ko04626), glutathione metabolism (ko00480), pentose and glucuronate interconversions (ko00040) (Figure 9D).

## Discussion

With the combination of genome sequencing with comparative analysis, enormous LSGs with potentially important functions have been identified in different species (Wilson et al., 2005; Zhang et al., 2007; Lin et al., 2010; Tsutsui, 2011), which motivated our genome wide exploration of LSGs within tea plant CSS. Before further analysis of LSGs, we need to identify LSGs first. Lin et al. identified 1324 LSGs in *A. thaliana* genome (Lin et al., 2010) and Ma et al. identified 3812 LSGs in wheat genome (Ma et al., 2020). Among this research, a grand sum of 1701 CSGs in the genome of CSS were identified, representing approximately 3.37% of the entire genome. This CSGs percentage was similar to the 4.9% found in *A. thaliana* (Lin et al., 2010) and 3.2% in rice (Yang et al., 2009). Since we used the genomes of published homologous species to identify LSGs, the more abundant the genome data of reference species available, the more information we could annotate and the less the false positives would be, though the number of LSGs might decrease. Although there are still shortcomings in our currently available identification tools such as pseudogene exclusion, our study remains a vital step in exploring new genes in CSS genome, and the identification of CSGs will become more accurate.

Accumulating researches have showed that some characteristics of LSGs may be somewhat different compared to ECs in all species, such as gene size, length of protein, GC content and number of exons, mainly related to the mechanism of origin and evolutionary time of LSGs. To reveal whether these differences in genic characteristics exist between CSGs and ECs, the sequence structure of CSGs and ECs were compared and analyzed. The average size of LSGs is normally smaller than ECs (Campbell et al., 2007; Zhang et al., 2007; Cai and Petrov, 2010; Lin et al., 2010; Yang et al., 2013), our result in CSS also comply with this conclusion (Table 1). This phenomenon may be related to the fact that each CSG has fewer exons (Table 1). Besides, CSGs have shorter protein lengths (Table 1), consistent with the LSGs of other eukaryotes (Domazet-Loso, 2003; Campbell et al., 2007; Toll-Riera et al., 2009; Donoghue et al., 2011). One reason for such differences between CSGs and ECs may be that intronless genes can be created by retroposition, which has been shown to create a large number of LSGs in the zebrafish genome (Fu et al., 2010). Alternatively, this phenomenon may be a result of the “introns late” hypothesis, which suggests that the accretion of intron into the protein-coding genes is continuous during the evolution of eukaryotes (Koonin, 2006). As a result, younger genes have fewer exons. Furthermore, since LSGs are species specific, they generally have emerged in relatively recent years. In summary, these reasons may partly explain why LSGs have fewer numbers of exons per gene and why LSGs are shorter than ECs. On the other hand, CSGs has significantly higher GC content than ECs in CSS, consistent with the results in *Bombyx mori* (Sun et al., 2015) and zebrafish (Yang et al., 2013). This is consistent with the observation in previous studies that the enrichment of high GC content class usually occurs in genes lacking introns (Carels and Bernardi, 2000; Alexandrov et al., 2009). However, this property is not universal, the GC content of LSG in other species like *Triticeae* is lower than that of ECs (Ma et al., 2020). Differences in GC content are the result of a combination of factors such as the external environment and habits of organisms, and the possible mechanisms responsible for these significant differences still need further study (Carels and Bernardi, 2000; Galtier et al., 2001; Halder et al., 2017). The isoelectric point has been considered to alter the protein function and indirectly reflect the species-specific adaption made in response to the variable environment (Nandi et al., 2005). In this study, the isoelectric points of CSGs were found higher than those of ECs and the difference can indirectly reflect the species-specific applicability of CSS to the environment.

The mechanisms of the origin of LSGs are vital for explaining the origin and evolution of new phenotypes and ultimately the genetic basis of biodiversity. There are four main mechanisms explaining for the origin of LSGs including gene duplication, transposon pattern, gene overlap, and *de novo* origin (Kaessmann, 2010; Long et al., 2013), among which gene duplication was considered to be most predominant (Long et al., 2003; Kaessmann, 2010; Tautz and Domazet-Loso, 2011; Wissler et al., 2013). Tautz believed that LSGs are formed by sequence variation after gene replication, and because of the acceleration of evolution, this gene loses its sequence similarity with other species genes, and thus LSGs appear (Opazo and Storz, 2008; Tautz and Domazet-Loso, 2011; Kondrashov, 2012). In this study, we found that 971 CSGs in CSS were derived from gene duplication, occupying 57.08% of the total CSGs. We evaluated the duplication time of CSGs using *Ks* peaks, and the result showed concordance with the synchronization of the two WGD events in the CSS genome (Wei et al., 2018). In CSS genome, gene duplication had brought large impact on the evolution of genes associated with the biosynthesis of secondary metabolites that are essential for tea aroma and flavor, such as genes involved in the catechin biosynthesis pathway were mostly generated by gene duplication.

Due to the rapid development of sequencing technology, the study of LSGs is now no longer limited to sequence structure but exploring gene function. RNA-Seq is an effective way to characterize the expression schemas of CSGs among various tissues (Wang et al., 2009). Studies have shown that there is difference in the expression of LSGs in different tissues, usually with higher expression in the reproductive system in animals (Begun et al., 2007; Chen et al., 2020) and also in plant tissues such as mature pollen (Wu et al., 2014) and callus (Xu et al., 2015). In this study, 212 genes were found to have significant tissue specific expression. There were 17, 32 and 29 CSGs expressed only in reproductive organs including apical bud, flowers and fruits, respectively, and 21, 18, 28, 25, and 42 genes in trophic organs including young leaves, mature leaves, old leaves, stems and roots, respectively (Table 2), implying that most CSGs play an important role in reproductive development. Besides, some LSGs have been reported to be important for tackling with extreme environmental conditions like cold, drought, heat and salt stress according to previous studies (Bosch et al., 2009; Yang et al., 2009; Donoghue et al., 2011). For CSS, both salinity and drought constitute severe challenges that significantly affect the production and qualities of CSS. We here checked the expression of CSGs under salt and drought stress, and observed that 12 genes had been stimulated, indicating that these 12 stress-responsive CSGs may be related to adaption to the extreme environmental conditions (Supplementary Table S6). CSS0030246, CSS0002298, CSS0018115, CSS0048226, and CSS0006611 were down regulated under 24 h salt stress. CSS0030246 was down regulated under 48 h salt stress. CSS0030939 and CSS0038744 were down regulated under 72 h salt stress. CSS0040193 was up regulated under 72 h salt stress (Supplementary Table S6). Over-expression of CSS0040193 may be associated with the tolerance of CSS to salinity. At the same time, CSS0002298, CSS0023764, CSS0046868, CSS0005736 and CSS0027450 were down regulated under 24 h drought stress. CSS0030246 and CSS0030939 were down regulated under 48 and 72 h drought stress, respectively (Supplementary Table S6). Interestingly, CSS0030246, CSS0002298 and CSS0030939 responded to both salt and drought stress, which may be candidates for further studying environmental adaptation in CSS.

Since having no homologous genes related in other species, the possible expression characteristics and functions of CSGs cannot be inferred by homology comparisons. However, we can infer the possible biological processes involved in CSGs by means of the co-expressed gene modules. In this study, we identified 18 CSGs in 4 tissue-specific modules with WGCNA (Supplementary Table S8), and identified that these co-expression gene modules were predominantly involved in phenylalanine biosynthesis, biosynthesis of amino acids, pentose phosphate pathway, photosynthesis and carbon fixation in photosynthetic organisms with KEGG analysis (Figure 9). Catechins are the main components of polyphenolic substances in tea leaves, which determine the unique aroma and flavor of tea. At the same time, the biological activity of catechins is of great significance to the prevention of various diseases and human health, such as the suppression of postprandial hypertriacylglycerolemia (Ikeda et al., 2005) and the prevention and therapy of cancer (Yiannakopoulou, 2014). Catechins are synthesized by a series of complex metabolic pathways, notably the flavonoids synthesis pathway, the pentose phosphate pathway and the shikimic acid pathway (Eungwanichayapant and Popluechai, 2009). The phenylpropane pathway is the starting pathway for flavonoids metabolism in plants, and the phenylpropane pathway uses phenylalanine as starting substrate(Lepiniec et al., 2006), indicating that the CSGs we identified play a crucial role in the synthesis of catechins and the formation of the specific flavor of CSS. In addition, photosynthesis not only determines the growth and productivity of plants, but also has a great impact on secondary metabolic pathways. Studies have shown that the biosynthesis of catechins in tea plants is regulated by light and its content is negatively correlated with chlorophyll concentration (Li et al., 2016; Hwa et al., 2019). The higher the chlorophyll content under low light, the lower the catechin content (Li et al., 2016; Hwa et al., 2019). In conclusion, CSGs involved in photosynthesis and carbon fixation are closely related to the productivity and quality of CSS.

## Conclusion

In this study, we identified 1701 CSGs from the CSS genome, accounting for 3.37% of the genome. Through structural characterizations analysis, we found that CSGs had shorter protein length, higher GC content and isoelectric point compared to ECs. Analysis of the origin of 1701 CSGs showed that 971 CSGs were derived from gene duplication, making up 57.08% of total CSGs. Besides, most CSGs were found mainly localized in the nucleus, extracellular space and chloroplasts. Gene expression analysis revealed tissue-specific expression of CSGs. The results of WGCNA showed that CSGs were mainly involved in pathways such as phenylalanine, tyrosine and tryptophan biosynthesis, pentose phosphate pathway, biosynthesis of amino acids, photosynthesis and carbon fixation. In addition, the expression of some CSGs was associated with stress tolerance. In conclusion, this study has provided a basis for studying the specific genetic resources of tea and provides some clues for future interpretation of the role played by tea LSGs in tea-specific features.

## Data Availability

The original contributions presented in the study are included in the article/Supplementary Material, further inquiries can be directed to the corresponding author.
